# Oral Amyloidosis Secondary to Tuberculosis in an Adult Patient: A Report of a Rare Case

**DOI:** 10.7759/cureus.91852

**Published:** 2025-09-08

**Authors:** Nyl Fumero, Maria G Gamboa, Michelle Jaimes, Melani Meza, Mariana Villarroel-Dorrego

**Affiliations:** 1 Faculty of Dentistry, Central University of Venezuela, Caracas, VEN; 2 Oral and Maxillofacial Surgery, General Hospital of the East "Dr. Domingo Luciani", Caracas, VEN; 3 Dental Research Institute, Central University of Venezuela, Caracas, VEN

**Keywords:** aa amyloid protein, birefringence, chronic inflammatory process, congo red stain, secondary amyloidosis, tuberculosis

## Abstract

Oral amyloidosis involving the lateral border of the tongue secondary to tuberculosis is a rare clinical finding. We present the case of a 35-year-old woman with presumed amyloidosis secondary to tuberculosis after increased tongue volume and irregularly shaped lateral edges were noticed. The present study was motivated by the correlation between tuberculosis and its involvement as a trigger in the appearance of amyloidosis, which, in this case, presents with oral manifestations. This retrospective case report describes a patient whose incisional biopsy confirmed oral amyloidosis secondary to tuberculosis. The patient had a history of infectious diseases such as toxoplasmosis, histoplasmosis, cytomegalovirus, Epstein-Barr virus, and tuberculosis. The latter influenced the development of the amyloidosis process, suggesting an underlying immunological compromise that was not evaluated. The amyloidosis treatment begins with identifying the amyloid protein involved; since this is a serum amyloid A (SAA) protein, this finding is then related to the presence of an underlying chronic infectious inflammatory pathology.

## Introduction

Amyloidosis is defined as a systemic disease characterized by extracellular deposits of amyloid protein fibers, which are formed by an amyloid precursor protein that undergoes abnormal folding in one or more organs, which stand out for their ability to be identified under an optical microscope by staining with Congo red, which is the gold standard staining for the demonstration of amyloid fibers in tissues, observing under polarized light an apple-green-like birefringence. It affects the oral cavity in 40% of cases, contemplating macroglossia, which is manifested by a diffuse increase in the size of the tongue with loss of elasticity and accumulations of amyloid material forming nodular lesions that can be distributed at the level of the tongue, salivary glands, and lips. This condition is progressive, giving rise to a generalized systemic involvement, which usually ends up taking the patient's life [1].

The incidence of amyloidosis in the population is eight people per million inhabitants per year, and it mainly affects women. The amyloid protein fibers most frequently involved in cases of amyloidosis are AL amyloid fibers (immunoglobulin light chain fibers) associated with primary amyloid deposits and AA amyloid fibers (serum amyloid A (SAA)) associated with secondary amyloid deposits linked to chronic infectious processes. Identifying the type of amyloid fiber present is essential for developing the correct treatment approach [2-5].

Secondary amyloidosis is usually associated with pulmonary tuberculosis, is the most frequently found form of amyloidosis, and presents a nonspecific clinical pattern. For its diagnosis, an incisional biopsy of the affected tissue is performed, processed with Congo red stain, and studied under a microscope with polarized light [2].

Tuberculosis is an infectious bacterial disease caused by *Mycobacterium tuberculosis*, which is transmitted between humans through the respiratory route and most commonly affects the lungs; however, this disease can damage any tissue. It can remain in an infected individual for many years in a latent state and then activate and generate a pathological action, which is why it is considered a chronic infectious process [3].

Latent tuberculosis can progress to active disease at any time, but the risk is highest during the first year after infection. People with HIV/AIDS or immunocompromised individuals are at increased risk of developing active tuberculosis, regardless of the time elapsed since the initial infection [3].

## Case presentation

**Figure 1:**
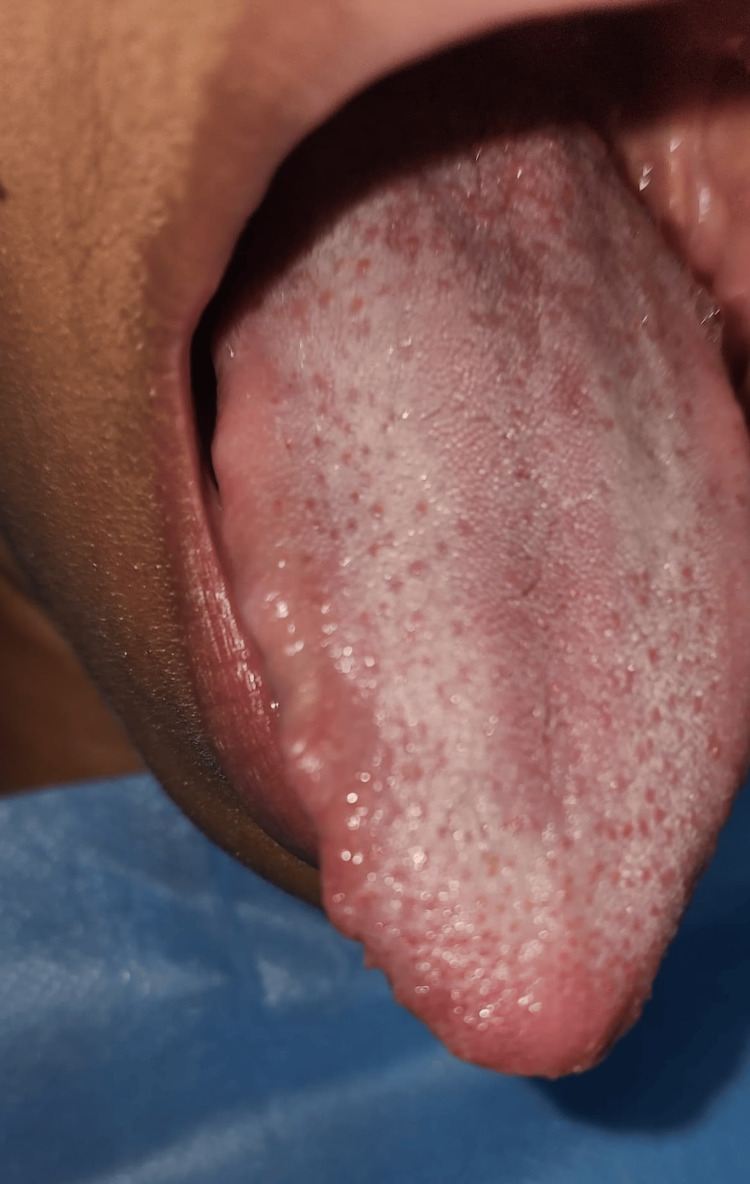
Clinical image of the tongue with increased volume and irregularly shaped lateral edges.

**Figure 2:**
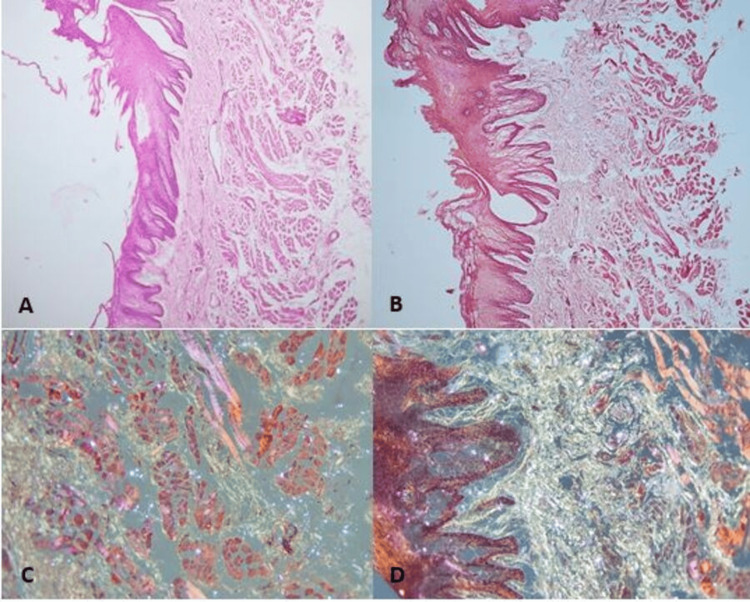
Microphotograph of the stained sample of oral mucosa. (A) 4× microphotograph with hematoxylin-eosin staining. (B) 4× microphotograph with Congo red staining. (C and D) 10× microphotograph with Congo red staining under polarized light. Note the apple-green color of the areas occupied by the amyloid protein.

A 35-year-old female HIV-negative patient from Venezuela with presumed amyloidosis and a medical history of pulmonary tuberculosis diagnosed in December 2022, toxoplasmosis, cytomegalovirus and Epstein-Barr virus infections, and granulomatous adenitis secondary to histoplasmosis presented with a one-year history of pain, burning sensation in the right lateral border of the tongue, and increased volume and irregularly shaped lateral edges (Figure 1). These findings were assessed at the Oral and Maxillofacial Surgery Department of the General Hospital of the East "Dr. Domingo Luciani" in February 2024 and were subsequently evaluated further. The presumptive diagnosis was oral amyloidosis secondary to tuberculosis. Preoperative hematological examinations and the performance of an incisional biopsy were indicated. The obtained sample of oral mucosa was processed, stained with hematoxylin-eosin and subsequently with Congo red stain to be examined under an optical microscope with conventional light, which resulted in a fragment of oral mucosa lined by normal-looking stratified squamous epithelium, discrete deposits of an amorphous, acellular, eosinophilic, fibrillary material in the corium and underlying striated muscle tissue, and foci of chronic inflammation, with no evidence of malignancy, and later observed with polarized light, where the normal-looking stratified squamous epithelium lining, the red-stained muscle fibers, and the deposits of eosinophilic material were observed to be strongly stained, appearing apple-green (Figure 2). Days after the intervention, a sialometry study was performed due to dry mouth sensation. The patient was prescribed ambroxol hydrochloride, chamomile, and linseed mouthwash to manage symptoms. As symptoms persisted without improvement, 810/980 nm diode laser therapy (EVO Ultradent©, South Jordan, UT, USA) at a power of 0.2 W was conducted to stimulate the major salivary glands in addition to the topical use of electrolyzed water three times a day for 15 days.

One year after treatment, the patient is pain-free and does not complain of dry mouth, and her tongue remains the same size and consistency.

## Discussion

Amyloidosis is a rare disease characterized by the extracellular deposition of insoluble fibrils composed of irregularly grouped proteins in various organs and tissues [4].

It is classified as AL amyloidosis or primary amyloidosis, AF or familial amyloidosis, ATTRwt or wild-type systemic amyloidosis, and AA or secondary amyloidosis [2]. Secondary amyloidosis is caused by the sustained production of a plasma protein known as an acute-phase reactant, the production of which is exacerbated in chronic inflammatory processes. The acute-phase reactant present in secondary amyloidosis is known as SAA, the synthesis of which occurs mainly in the liver and secondarily in extrahepatic cells (adipocytes, synovial membrane, arterial wall). This response occurs due to the elevation of plasma cytokines such as IL-1, IL-6, and tumor necrosis factor-α, characteristic of patients with chronic inflammatory diseases [5-9].

This form of secondary amyloidosis has been associated with chronic infectious processes, including tuberculosis, which is the most frequent cause of the development of secondary amyloidosis, as well as chronic inflammatory processes such as bronchiectasis, rheumatoid arthritis, inflammatory bowel disease, and chronic osteomyelitis [2,7,10,11].

The literature contains only a few reports of secondary amyloidosis affecting the tongue, and to the authors' knowledge, none have described a case following pulmonary tuberculosis. Therefore, this case adds new evidence to the existing literature by documenting a rare oral presentation linked to a well-known systemic disease. Previous studies have primarily reported oral involvement in the context of systemic amyloidosis [6,9], whereas the present patient showed localized amyloid deposition despite having a recognized systemic trigger. This finding aligns with the established role of chronic infections such as tuberculosis in the development of amyloidosis, but differs in its absence of multi-organ involvement [7]. By highlighting this atypical presentation, this report reinforces the known pathophysiological mechanisms of secondary amyloidosis and broadens the clinical spectrum of its manifestations.

The patient's prognosis will be closely related to the clinical involvement of organs by this disease. In cases of localized amyloidosis, where there is no significant systemic involvement, amyloid deposits will present in the nasopharyngeal, pulmonary, cutaneous, and lymph node regions; however, it will not lead to cardiomyopathy, proteinuria-induced nephropathy, or hepatomegaly, since amyloid deposits are not found at a generalized systemic level [7]. As long as the origin of the chronic infectious or inflammatory process can be controlled and/or eradicated, this will result in a progressive decrease in SAA deposits, thus giving favorable expectations of evolution for the treated patient [6,12].

The prognosis for this patient is good due to four major factors: early diagnosis of the disease, localization of protein deposits that do not compromise organ function, multidisciplinary management that results in appropriate treatment, and the patient's willingness to attend her appointments and obtain the resources for her treatment [7].

## Conclusions

This case highlights the importance of considering secondary amyloidosis in the differential diagnosis of persistent oral lesions, particularly on the tongue, even in the absence of systemic symptoms, as this oral condition is exceptionally rare when related to tuberculosis. Early recognition and specific treatment of the underlying infectious process are crucial to prevent further systemic involvement and improve patient prognosis. It is essential to emphasize that the diagnosis and treatment of oral amyloidosis must be performed by a multidisciplinary team, including dentists and physicians, to achieve adequate disease management.
